# Performance Analysis of Cold-Mixed Integrated Semi-Flexible Pavement Mixtures

**DOI:** 10.3390/ma19091757

**Published:** 2026-04-25

**Authors:** Qinxue Pan, Yang Zhao, Milkos Borges Cabrera, Jia Hu, Xiaojin Song, Xudong Zha, Yuting Tan

**Affiliations:** 1 School of Transportation, Changsha University of Science & Technology, Changsha 410114, China; milkos_csust@126.com (M.B.C.); hj0805hj@163.com (J.H.); zhaxd410@163.com (X.Z.); 2 National Key Laboratory of Green and Long-Life Road Engineering in Extreme Environment (Changsha), Changsha University of Science & Technology, Changsha 410114, China; 3 National Engineering Research Center of Highway Maintenance Technology, Changsha University of Science & Technology, Changsha 410114, China; 4 Zhongteng Zhixin Technology (Hunan) Co., Ltd., Changsha 410036, China; sxjmail@126.com; 5 Chengnan College, Changsha University of Science & Technology, Changsha 410076, China; 15973107992@163.com

**Keywords:** semi-flexible pavement, cold-mixed integrated, pavement performance, microscopic analysis

## Abstract

To address the issues of high energy consumption and unstable construction quality caused by high-temperature heating during the preparation of traditional hot-mixed/grouted semi-flexible pavement (SFP) mixtures, a cold-mixed integrated (CMI) process was proposed. In addition, the material composition of the mixtures was optimized. The effects of the preparation process and binder type on the high- and low-temperature performance, water stability, and fatigue performance were then analyzed. Furthermore, the microstructural characteristics of the semi-flexible mixture were also investigated. The results indicated that the CMI process facilitated the formation and uniform distribution of calcium silicate hydrate (C-S-H), enhanced the binder’s ability to encapsulate aggregates and fill skeletal voids, significantly reduced the mixture’s void ratio, and improved its pavement performance. The proposed procedure was a means of enhancing high-temperature stability and fatigue life (an increase of 80% and 200 times compared to the hot-mixed/grouted (HMG) process, and 5 times and 300 times compared to AC-13, respectively). Compared with the HMG process, the CMI process offered greater advantages in enhancing the high-temperature stability and fatigue resistance of the mixture, particularly when using SBS-modified asphalt, where fatigue performance exhibited an order-of-magnitude improvement. Furthermore, while SBS modification could improve the road performance of SFP materials, mixtures prepared with SBS-modified emulsified asphalt demonstrated more significant enhancements in high-temperature stability and fatigue resistance, approximately 2 times and 10 times higher than SBS-modified mixtures, respectively. The addition of styrene–acrylic emulsion (SAE) could further enhance the low-temperature crack resistance by approximately 7%. The research results can provide a reference for the development and application of preparation processes for semi-flexible mixtures.

## 1. Introduction

With the continuous increase in traffic loads and the complexity of service environments, novel and durable pavement materials that combine excellent service performance with environmental benefits are an important target to be accomplished [1,2,3]. At the same time, the large-scale consumption of asphalt and mineral aggregates has imposed significant pressure on traditional pavement material systems in terms of resources and carbon emissions in road engineering [4,5].

The SFP mixture was formed by combining a high-pore-ratio asphalt mixture with a cement-based paste, creating a multiphase structural system that combined rigidity and toughness. It exhibited excellent rut resistance and structural stability under heavy traffic conditions [6,7]. However, the performance of this type of material was fundamentally limited by the pore structure, paste filling, and the intrinsic connection within the interface transition zone. Therefore, the quality of the preparation process significantly affected the pavement performance of the mixture. Current research has mainly focused on optimizing the composition of the grouting material and designing the base gradation [8,9]. The relevant results indicated that the type of paste and asphalt modification method could significantly affect the early strength and durability of the mixture [10,11]. The addition of emulsified asphalt and polymers helped improve the flexibility and crack resistance of the material. Microscopic analysis also revealed that the cement hydration products and asphalt intermingled [12,13], forming a composite structure with a spatial network, but the porosity remained large, and the compactness of the mixture still needed to be further improved. However, in general, the existing research had mainly focused on the material composition level and lacked systematic characterization of the preparation process of the mixture, its internal structure, and its intrinsic correlation with pavement performance.

Currently, the HMG process is the most commonly used technical method for SFP mixtures. Its core lay in achieving the penetration and filling of the slurry through reserved connected pores. However, the mixtures prepared by this process were significantly affected by their internal pore structure characteristics. On the one hand, too low a porosity rate limited slurry penetration and resulted in insufficient filling; on the other hand, an excessively high porosity rate reduced the stability of the skeleton structure [14]. Therefore, in engineering, the void ratio of the base asphalt mixture was usually maintained at approximately 18%, and the post-injection void ratio was controlled within 5% [15]. This empirical control range was difficult to balance the stability of the mixture’s road performance under different material systems and construction conditions, and the HMG process was significantly affected by temperature, rheological properties, and construction disturbances, which could easily lead to uneven filling, local defects, and performance dispersion [16]. In addition, the traditional injection-based process required multiple procedures such as paving, cooling, injection, and curing, which not only had a long construction period but also made the interface formation process of the mixture uncontrollable, further increasing structural non-uniformity [17].

From the perspective of the formation mechanism, in the grouting process, the construction of the mixture skeleton and the filling of the slurry were separated in time and space. After the asphalt mixture skeleton was formed, the slurry entered the interior of the pores through osmosis. This process had high requirements for the fluidity of the slurry and the connectivity of the pores. Otherwise, it was prone to cause uneven filling degree and distribution state, thereby affecting the formation of the interface transition zone and the uniformity of the pore structure. This feature, to some extent, limits the overall density and performance stability of the material structure. Therefore, it was necessary to explore a new preparation process that could achieve the coordinated distribution of components and the synchronous construction of the structure during the mixing stage.

Based on this, this paper proposed a cold mix integrated (CMI) method for preparing semi-flexible mixed materials. By achieving uniform distribution of the asphalt phase and the cement-based paste during the mixing stage, this method avoided the problems of uneven penetration and filling during the traditional grouting process. Subsequently, the differences in pavement performance between the cold-mixed integrated and hot-mixed grouted (Hot-Mix Grouting, HMG) were compared and analyzed. The influence of different binder types (base asphalt, 70# emulsified asphalt, SBS modified asphalt, and its emulsified asphalt) on the pavement performance and micro-performance of the mixture was also studied. At the same time, styrene–acrylic emulsion (SAE) was introduced to reveal its improvement effect on the comprehensive performance of the mixture. The research results could provide a theoretical basis for the process optimization and performance enhancement of semi-flexible pavement materials.

## 2. Raw Materials and Experimental Methods

### 2.1. Material Composition

#### 2.1.1. Raw Materials

This study was conducted to investigate the influence of asphalt types on semi-flexible pavement materials. The asphalt selected comprised matrix asphalt, modified emulsified asphalt, SBS-modified asphalt, and SBS-modified emulsified asphalt—were all produced by Hunan Baoli Asphalt Co., Ltd. (Changsha, China). Table 1, Table 2, Table 3 and Table 4 show the technical specifications of the asphalt and the styrene–acrylic emulsion, styrene–acrylic emulsion produced by Shandong Dongrun Chemical Industry (Yueyang, China). The test showed that all its indexes met the requirements of JTG 3410-2025 [18].

#### 2.1.2. Cement Mortar

The P.C. 42.5 cement produced by Hunan Southern Cement Co., Ltd. (Changsha, China), an orthogonal experimental design was adopted in this study to optimize the mix proportion of the composite-blended cement mortar. Different combinations of factors and levels were designed to analyze the influence of each factor on the workability and mechanical properties of the mortar, based on which the optimal parameter combination was selected. The final mix proportion was determined as follows: the water-to-binder ratio, sand-to-binder ratio, and silica fume-to-binder ratio were 0.6, 0.1, and 0.15, respectively, and the dosages of water, expansive agent, and early-strength agent were 0.5%, 1%, and 1%, respectively. The corresponding performance indices are listed in Table 5. The experimental results indicated that the optimized cement mortar exhibited good fluidity and met the specification requirements, while also showing a relatively low compressive-to-flexural strength ratio and relatively high mechanical strength.

#### 2.1.3. Gradation Design of Grouted Base Asphalt Mixture

**Figure 1:**
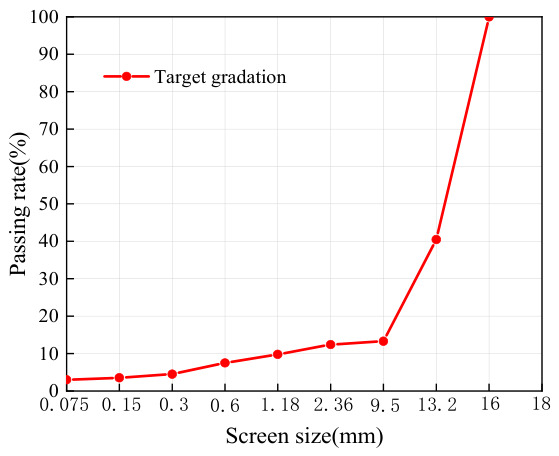
Gradation design results of matrix asphalt concrete [19].

In this study, the gradation design of the matrix large-void asphalt mixture was carried out using the volumetric method. The selection of 24% as the target void content is based on literature research and our earlier experimental results. Specifically, when the void content of the asphalt mixture is around 24%, the prepared grouting semi-flexible mixture exhibits the best road performance [20]. Based on this finding, the air void content in this study was set at 24%. The material composition and volumetric parameters of the matrix asphalt mixture are presented in Figure 1.

#### 2.1.4. Cold-Mixed Integrated Semi-Flexible Pavement Mixtures Design

(1) Gradation Design

**Figure 2:**
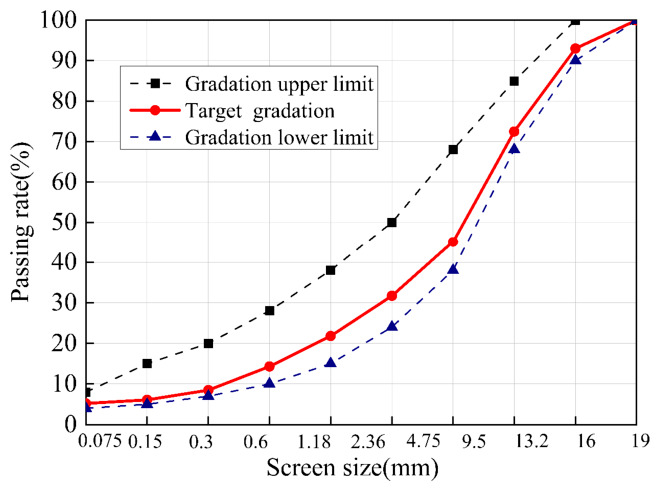
Gradation design results of matrix asphalt concrete.

The cold-mixed integrated semi-flexible pavement materials adopted the optimal cement mortar mix ratio from Section 2.1.2. According to China’s JTGF40-2004 [21], the mix design for the AC-13 was conducted following the gradation requirements. Figure 2 shows the gradation curve.

(2) Optimal Emulsified Asphalt Content

**Figure 3:**
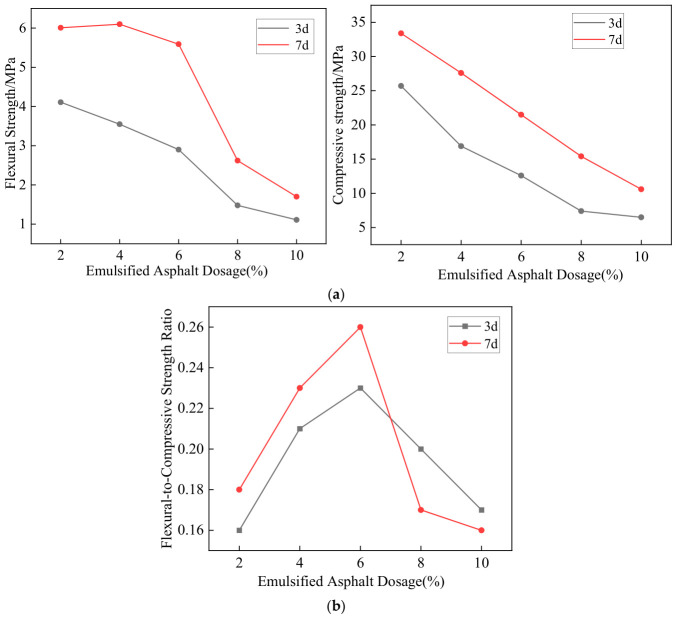
Research on the mechanical properties of emulsified asphalt under different dosages. (**a**) Compressive strength and flexural strength. (**b**) Flexural-to-compressive strength ratio.

In cold-mixed integrated semi-flexible pavement mixtures, cement mortar combined with emulsified asphalt served as the binder among aggregates. Flexural and compressive strengths were used as evaluation indices to determine the optimum emulsified asphalt content. In preliminary tests, 2%, 4%, 6%, 8%, and 10% emulsified asphalt (by mass) were added to the cement mortar. The flexural and compressive strengths were then measured according to China’s JTG 3420-2020 [22]. Figure 3 shows the results.

Figure 3 shows that as the emulsified asphalt content increases, the compressive and flexural strengths of the specimens at both 3 d and 7 d generally decrease, indicating that the addition of emulsified asphalt weakens the material’s mechanical properties to some extent while helping alleviate its brittleness. In terms of curing age, both compressive and flexural strengths of all specimen groups at 7 d are significantly higher than those at 3 d, indicating that as the curing time is prolonged, the cement hydration reaction proceeds further, the paste structure becomes denser, and the overall mechanical properties of the material are improved. When the emulsified asphalt content is 6%, the flexural-to-compressive strength ratio reaches its maximum value, indicating that the improvement in material toughness is most significant at this dosage. Moreover, at 7 d, the compressive strength still meets the specification requirement (≥20 MPa). Considering mechanical properties, improvements in brittleness, and specification requirements, 6% emulsified asphalt was determined to be the design dosage for the CMI-SFP mixture.

### 2.2. Experimental Methods

#### 2.2.1. Test Scheme

**Figure 4:**
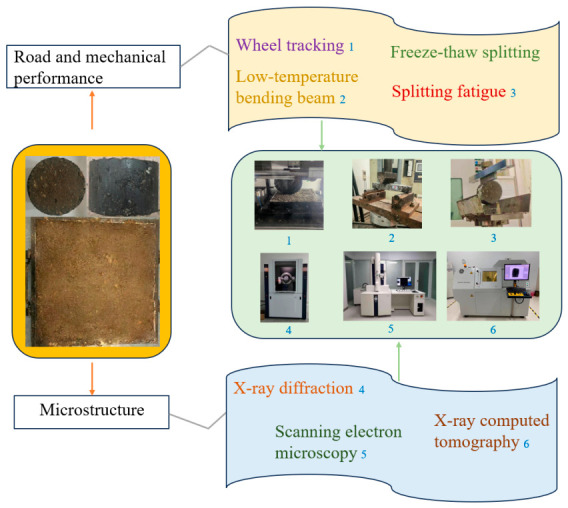
Testing procedure for this study.

To evaluate the suitability of the mixture for the surface course, this study involved both mechanical properties and microscopic performance tests for different pavement materials. Each test was conducted using three replicate samples, and the results obtained were based on the average values derived from these tests. Figure 4 shows the testing process of this study. Table 6 lists the relevant abbreviations.

#### 2.2.2. Test Method

(1) Road performance tests

This study investigated the road performance of semi-flexible mixtures and AC-13 asphalt mixtures prepared using different processes. High-temperature performance, low-temperature performance, water stability, and fatigue performance were evaluated in accordance with China’s JTG E20-2011 [17].

High-temperature performance was evaluated using the rutting test. Rutting plate specimens (300 mm × 300 mm × 50 mm) were prepared according to the specifications and cured for 7 days in a standard curing room at 20 ± 3 °C and relative humidity above 90%. The rutting test was performed at 60 °C with a wheel pressure of 0.7 MPa after the specimens were conditioned at the test temperature for 5 h. Low-temperature performance was assessed using the small-beam bending test. After curing, the rutting plate specimens were cut into prismatic beams with dimensions of 250 mm × 30 mm × 35 mm. These beams were conditioned at −10 °C for 5 h before being tested on an MTS Landmark multifunctional testing machine (MTS Systems Corporation, Eden Prairie, MN, USA). The test was conducted with a 200 mm span and a constant loading rate of 50 mm/min.

Water stability was evaluated using the freeze–thaw splitting test. Standard Marshall specimens (101.6 mm × 63.5 mm) were prepared and cured for 7 days. The splitting tensile strength ratio was determined by comparing specimens subjected to freeze–thaw cycles with those without freeze–thaw treatment. Fatigue performance was evaluated using the splitting fatigue test. Standard Marshall specimens were tested using an MTS Landmark multifunctional material testing machine under stress-controlled loading. A continuous semi-elliptical load with a frequency of 10 Hz was applied at a test temperature of 15 °C.

(2) Microstructure tests

The hydration products of the cement binder were characterized using X-ray diffraction (XRD, Bruker AXS GmbH, Karlsruhe, Germany). XRD measurements were performed using a Bruker diffractometer (Germany) with a scanning range of 3–90°, a scanning rate of 8°/min, and a step size of 0.02°. The morphology of hydration products and the gel structure of SFPM were observed using scanning electron microscopy (SEM, Hitachi High-Tech, Tokyo, Japan). After curing in a standard curing room for 7 days, semi-flexible pavement materials prepared by the two processes were cut into small rectangular specimens with dimensions of 0.5 cm × 0.5 cm × 0.1 cm, and the specimen surfaces were gold-coated prior to SEM observation. The pore structure of SFPM was further investigated using X-ray computed tomography (CT, Waygate Technologies, Wunstorf, Germany). Cylindrical specimens (100 ± 2 mm in height and 100 ± 2 mm in diameter) prepared using an SGC rotary compaction machine were scanned using a GE Vtomexs CT system (Waygate Technologies, Wunstorf, Germany). With a layer spacing of 0.08 mm, approximately 1000 slices were acquired for each specimen, and pore structure parameters were obtained through image processing and quantitative analysis.

## 3. Preparation Process and Specimen Fabrication

### 3.1. Hot-Mixed/Grouted Semi-Flexible Pavement Material Preparation

**Figure 5:**
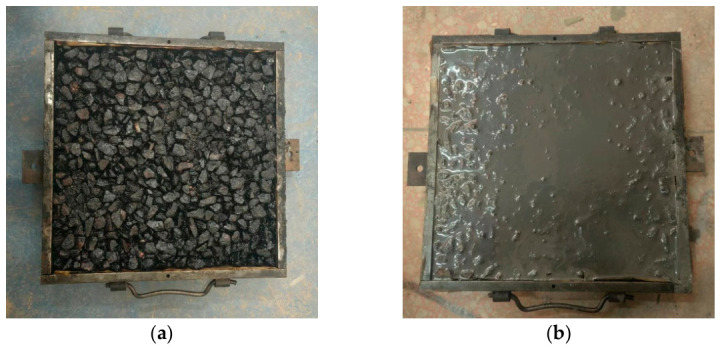
(**a**) Matrix Porous Asphalt Concrete. (**b**) HMG-SFP material.

Based on the cement mortar mix proportion determined in Section 2.1.2 and the optimum asphalt–aggregate ratio of 3.5% obtained from the preliminary tests, the hot-mixed/grouted semi-flexible mixture was prepared according to the method in the Chinese standard DB37/T 4681-2023 [23]. After grouting, the specimens were cured in a standard curing chamber with a relative humidity of not less than 90% and a temperature of 20 ± 3 °C. Demolding was carried out after 3 days. When the designed air void content was 24%, the grouting rate reached 96.5%, observing the 85% standard requirement. Figure 5 shows the comparison diagram of the asphalt concrete slab specimen before and after grouting.

### 3.2. Preparation Process of Cold-Mixed Integrated Semi-Flexible Mixtures

The CMI semi-flexible pavement material was prepared using the cement mortar mix proportion determined in Section 2.1.2, as well as the gradation design and optimum emulsified asphalt content determined in Section 2.1.4. The mixing sequence and compaction method substantially affect the performance of semi-flexible pavement mixtures. Emulsified asphalt and cement can easily demulsify during mixing, potentially reducing pavement performance. To address this issue, this study compared two mixing sequences and two compaction levels and then optimized them to develop a more suitable preparation process for a semi-flexible pavement mixture.

**Figure 6:**
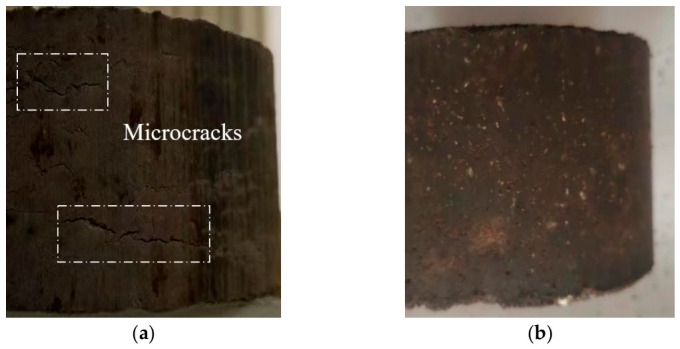
Standard Marshall specimens. (**a**) Method A. (**b**) Method B.

According to the mixing processes specified in the Chinese Standard JTG E20-2011 [17], two processes were designed. In Method A, the emulsified asphalt was first blended with cement to produce a cement emulsified asphalt mortar. This mortar was then mixed with aggregates. In Method B, the solid materials, such as cement, aggregates, and mineral filler, were uniformly mixed before being combined with liquid materials such as emulsified asphalt. The prepared specimens followed the standard Marshall dimensions (101.6 mm × 63.5 mm) and were compacted with 75 blows on one side and 50 blows on the other for comparison. Table 7 shows the results, and Figure 6 illustrates the specimens.

**Figure 7:**
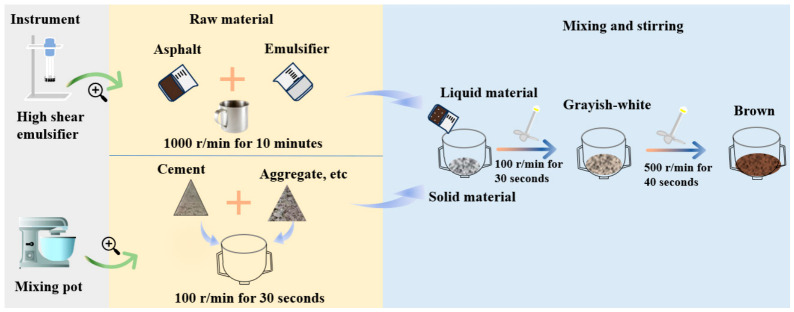
Preparation process for CMI-SFP mixtures.

Table 7 illustrates that when compaction times are 75 blows, the stability and void saturation of the semi-flexible pavement mixture increase, while the flow value and bulk density decrease. Due to the certain viscosity of the cement mortar at the early stage of forming, it filled the voids and contact areas of the aggregates, exerting a constraining effect on the rotation and rearrangement of the aggregate particles. Semi-flexible pavement materials exhibit higher compaction difficulty during the molding process compared to traditional asphalt mixtures. Increasing the number of compactions within a reasonable range helped to promote densification of the internal structure. Figure 6 shows that Marshall specimens prepared with Method A develop visible cracks (Dashed boxes highlight the cracks). The cracks appeared because, under the same water content, the emulsified asphalt first comes into contact with cement, causing premature demulsification. As a result, the cement emulsified asphalt mortar cannot fully coat the aggregates, which leads to crack formation. Therefore, this study adopted Method B for preparing the cold-mixed integrated semi-flexible pavement (CMI-SFP) mixtures. Figure 7 demonstrates the detailed process and includes the following steps:

(1) Add the asphalt and emulsifier into the shear mixer. Set the speed to 1000 r/min and mix for 10 min. This process provides sufficient shear while preventing premature demulsification. The process ensures uniform dispersion of the emulsifier and maintains the stability of the emulsified asphalt. Set the mixture aside for later use.

(2) Based on the preliminary tests, add cement, aggregates, mineral filler, and other solid materials into the planetary cement mortar mixer at the same time. Set the mixer to a low speed of 100 r/min and mix for 30 s until the materials are uniformly blended. This method helps prevent sand segregation and reduces equipment wear.

(3) To prevent liquid splashing, slowly add the prepared emulsified asphalt into the mixer running at 100 r/min and mix for 2 min. Then, increase the speed up to 500 r/min and continue mixing for 40 s. This procedure ensures that the emulsified asphalt fully coats the aggregate surfaces. During this process, the mixture’s color changes from light gray to brown.

(4) Compact the mixture with a Marshall compactor, applying 75 blows on each side. This produces standard Marshall specimens with dimensions of 101.6 mm × 63.5 mm.

## 4. Analysis of Mechanical Properties

This study compared the pavement performance of semi-flexible pavement mixtures AC-13 prepared with different binders and preparation processes. The results show that the performance of semi-flexible pavement mixtures depended on the asphalt type, the use of admixtures, and the preparation processes. Leading to significant differences.

### 4.1. High Temperature Stability

High-temperature performance was evaluated using the rutting test. This method was applied to systematically evaluate the effects of different preparation processes and material compositions on the high-temperature stability of semi-flexible pavement mixtures (SFP mixtures).

**Figure 8:**
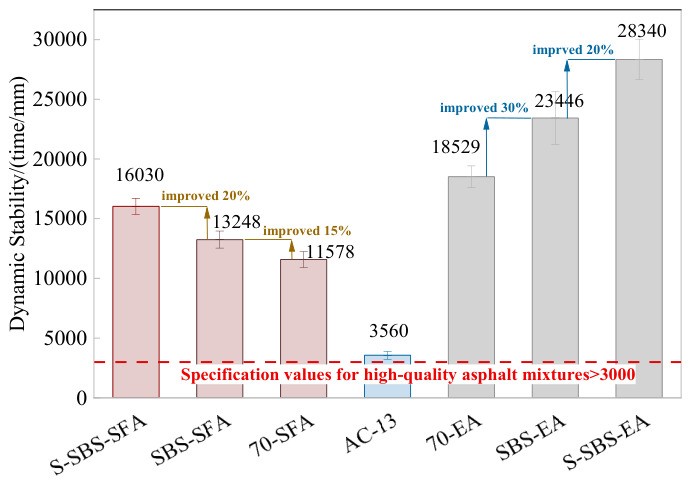
Dynamic stability of SFP mixtures.

Figure 8 shows that the dynamic stability of semi-flexible pavement mixtures increases by 15% when prepared with SBS-modified asphalt compared with 70# base asphalt. For conventional emulsified asphalt, using SBS-modified emulsified asphalt raises the dynamic stability by 30%. Compared to AC-13, semi-flexible mixtures prepared with SBS-modified asphalt and SBS-modified emulsified asphalt exhibit the most significant increase in dynamic stability, which is three times and six times higher, respectively. Emulsified SBS-modified asphalt also achieves nearly twice the improvement in high-temperature performance compared with SBS-modified asphalt alone. This finding is basically consistent with the findings of previous studies [24]. Overall, both SBS-modified asphalt and SBS-modified emulsified asphalt significantly enhance the high-temperature performance of SFP mixtures, with emulsified SBS-modified asphalt offering the best rutting resistance.

The test results demonstrate that adding SAE under the same preparation method increased dynamic stability by about 20%. This phenomenon is mainly attributed to the polymer network structure formed after the hardening of the styrene–acrylic emulsion, whose functional groups could physically entangle and chemically cross-link with the cement hydration products. As reported in previous studies, this interaction modified the material’s viscoelastic behavior, thereby enhancing its bonding and cohesive strength and effectively suppressing rheological deformation at high temperatures [25]. As a result, incorporating SAE further enhances the high-temperature performance of SFP mixtures.

Under both preparation methods, the dynamic stability of SFP mixtures is much higher than that of AC-13. The CMI process shows the best high-temperature performance. Specifically, the dynamic stability of 70-EA material is around 60% higher than that of 70-SFP material, SBS-EA material is about 80% higher than SBS-SFP material, and S-SBS-EA material is approximately 80% higher than S-SBS-SFP material. Compared with AC-13, S-SBS-EA material achieves a sevenfold increase. These results show that the CMI process improves the high-temperature stability of mixtures more effectively than the HMG process, and this advantage is superior when using modified asphalt.

### 4.2. Low-Temperature Cracking Resistance

A low-temperature bending test was conducted to evaluate the cracking resistance of asphalt mixtures and to compare the low-temperature performance of mixtures prepared by different processes.

**Figure 9:**
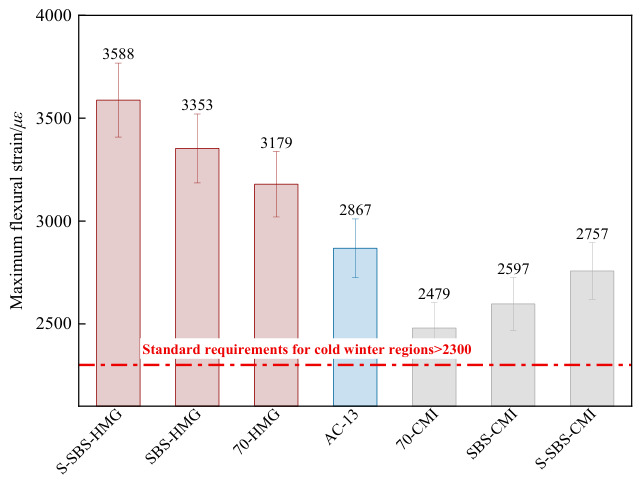
Maximum bending strain of SFP materials [21].

Figure 9 illustrates that SBS modification reduces asphalt brittleness at low temperatures, increasing the maximum flexural strain of SFP mixtures by about 5% compared with 70# base asphalt and its emulsified form. Compared with the AC-13, SFP mixtures prepared with SBS-modified asphalt show around a 25% increase in maximum flexural strain, while those with SBS-modified emulsified asphalt showed about a 10% reduction. These results indicate that SBS-modified asphalt can effectively enhance the low-temperature performance of the SFP mixtures. However, this improvement weakened after the SBS-modified asphalt emulsification; the SFP material’s performance is still better than that of the base asphalt. This trend is generally consistent with the influence of different asphalt types on the material properties reported in previous studies [26].

Incorporating SAE reduces the inherent rigidity of cement, thereby improving the low-temperature cracking resistance of SFP mixtures and increasing the maximum flexural strain by about 7%. Published studies have shown that during cement hydration, the emulsion formed a film that encapsulated Ca(OH)_2_ and suppressed the growth of significant crystalline phases, which helped relieve stress concentration and lower the risk of crack initiation and propagation under external loading [27].

Compared with the HMG process, the maximum flexural strain of CMI-SFP materials is about 20% lower. However, after adding SAE, their maximum flexural strain reaches values closer to those of AC-13 and meets specification requirements. This phenomenon shows that the CMI process is particularly suitable for regions exposed to severe rutting at high temperatures.

### 4.3. Water Stability

Freeze–thaw splitting tests were carried out to evaluate the water stability of semi-flexible pavement mixtures prepared by different processes.

**Figure 10:**
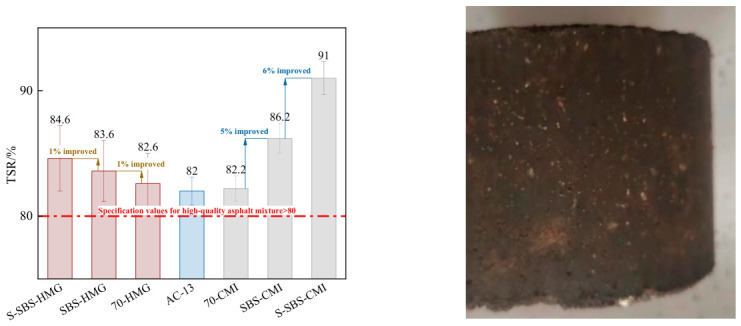
Freeze–thaw splitting residual strength ratio of SFP mixtures.

Figure 10 illustrates that the tensile strength ratio (TSR) of SBS-CMI materials is about 5% higher than that of the 70-CMI mixture and AC-13. In contrast, the SBS-HMG mixture shows smaller increases of around 1% and 2% compared with the 70-SFP mixture and AC-13. After emulsification, the TSR of SBS-modified asphalt rises to nearly five times the increase observed before emulsification. This indicates that both SBS modification and emulsification improve the water stability of the material, although the overall gain remains limited.

Styrene–acrylic emulsion possessed excellent film-forming and adhesive properties, forming a flexible, continuous polymer film between the asphalt–aggregate or asphalt-cement paste. This effectively inhibited water’s delamination effect on the binder, thereby reducing water damage to the interfacial structure. After incorporating styrene–acrylic emulsion, the TSR of the CMI-SFP mixture increased by approximately 6%, while the HMG-SFP mixture only improved by about 1%. This disparity may stem from the CMI process facilitating uniform dispersion of the styrene–acrylic emulsion. The emulsion can fully permeate and form a dense coating on the aggregate surface, significantly enhancing interfacial bonding and overall load-bearing capacity.

The TSR of the HMG-SFP materials exceeded 80%, showing good water stability. The CMI process further increased TSR, giving the mixtures superior water stability. Specifically, the TSR values of the 70-CMI mixture and the 70-HMG mixture were nearly the same. The SBS-CMI mixture was about 3% higher than the SBS-HMG mixture, the S-SBS-CMI mixture was around 7% higher than the S-SBS-HMG mixture, and the S-SBS-CMI mixture was almost 10% higher than AC-13. After adding SAE, the water stability of the CMI-SFP mixtures improved about six times more than that of the HMG-SFP mixtures. This phenomenon suggested that under the CMI process, the beneficial effect of SAE on enhancing water stability was more fully realized.

### 4.4. Fatigue Durability

**Figure 11:**
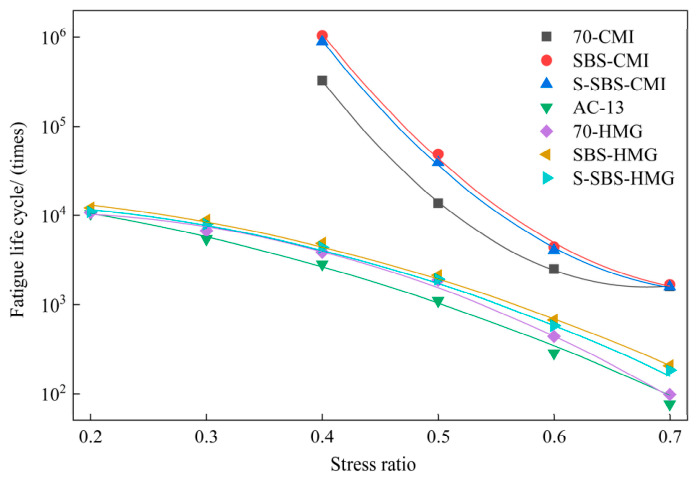
Relationship between fatigue life and stress ratio.

This study conducted splitting fatigue tests, and stress ratios for CMI-SFP mixtures were 0.4, 0.5, 0.6, and 0.7. For HMG-SFP mixtures, the ratios were 0.2, 0.3, 0.4, 0.5, 0.6, and 0.7. A regression fitting analysis was also performed to examine the relationship between the fatigue life of SFP materials and the applied stress ratio. Figure 11 illustrates the relationship between fatigue life and stress ratio.

Figure 11 shows that the fatigue life of pavement materials is strongly correlated with the stress ratio. Under the same conditions, mixtures prepared with SBS-modified asphalt and SBS-modified emulsified asphalt perform much better than those with 70# base asphalt and its emulsified form. At a stress ratio of 0.4, the fatigue lives of SBS-modified asphalt and SBS-modified emulsified asphalt increased by about 20% and 2 times, respectively, compared with AC-13, which are nearly 350 and 300 times higher. Among these results, SBS-modified emulsified asphalt shows the best fatigue resistance, almost 10 times greater than that of direct SBS modification. In light of previous studies, this phenomenon may be attributed to the emulsification of SBS-modified asphalt, which can significantly improve the interfacial bonding strength between the matrix asphalt and the cement paste, thereby enhancing resistance to crack propagation [28].

The addition of SAE had little effect on reducing fatigue life. At a stress ratio of 0.4, the fatigue life of SFP mixtures with SAE decreased by less than 10%. This result indicated that the flexible polymer formed after SAE film formation tended to fail under repeated loading, which weakened the fatigue resistance of SFP mixtures at all stress ratios.

At all stress ratios, the fatigue life of CMI-SFP materials was much higher than that of HMG-SFP mixtures and AC-13. At a stress ratio of 0.4, the 70-CMI mixture showed a fatigue life about 80 times greater than the 70-HMG material; SBS-CMI materials were about 210 times greater than SBS-HMG, and S-SBS-CMI material was nearly 200 times greater than S-SBS-HMG mixture, while also being about 300 times greater than AC-13. These results demonstrated that CMI significantly improves fatigue performance, especially in modified systems (SBS and S-SBS). When using SBS-modified asphalt, the fatigue performance of CMI-SFP mixtures improved by approximately one order of magnitude compared to HMG-SFP mixtures, maximizing the advantage of SBS-modified asphalt in enhancing the fatigue performance of semi-flexible mixtures.

## 5. Analysis of the Microstructure

### 5.1. XRD Diffraction Pattern Analysis

**Figure 12:**
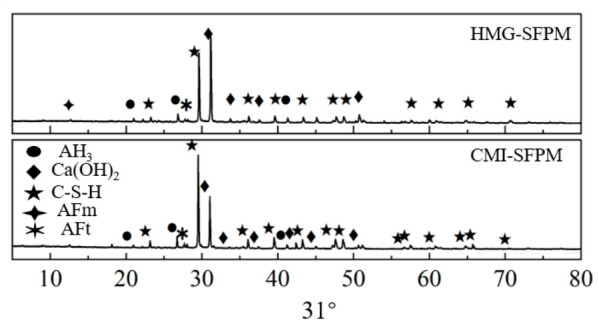
XRD diffraction patterns of cement mortar at different processes.

To analyze the hydration mechanism of mixtures prepared by different processes, X-ray diffraction (XRD) was used to study their hydration products and microstructure. XRD tests were carried out on HMG-SFP and CMI-SFP after 7 days of curing, and Figure 12 presents the results.

Figure 12 shows that the main hydration products of the cement mortar in both preparation processes are calcium silicate hydrate (C-S-H), calcium hydroxide [Ca(OH)_2_], calcium aluminate hydrates (C-A-H), ettringite (AFt), monosulfate (AFm), and aluminum hydroxide (AH_3_). This study did not detect new hydration phases. This indicated that the interaction between hydration products and asphalt in the preparation processes was governed by physical bonding and filling rather than chemical reactions.

Simultaneously, it was observed that the SFP material under the CMI process had a significantly lower peak intensity of Ca(OH)_2_ at a diffraction angle of 31° compared to that of the HMG process. Moreover, the C-S-H gel-related characteristics were more prominent, indicating that this process effectively promoted the further hydration reaction of low-strength Ca(OH)_2_, generating more high-strength hydration products dominated by C-S-H. As the content of C-S-H gel increased, the continuity and density of the inorganic bonding system within the material were significantly enhanced, thereby improving the structural stability and bearing capacity of the material and contributing to its high-temperature stability [29]. Conversely, as the overall stiffness of the inorganic bonding material increased, the deformation capacity of the inorganic-organic composite bonding system of the semi-flexible mixture weakened, leading to a decrease in its low-temperature crack resistance.

### 5.2. Scanning Electron Microscopy (SEM) Phase Analysis

**Figure 13:**
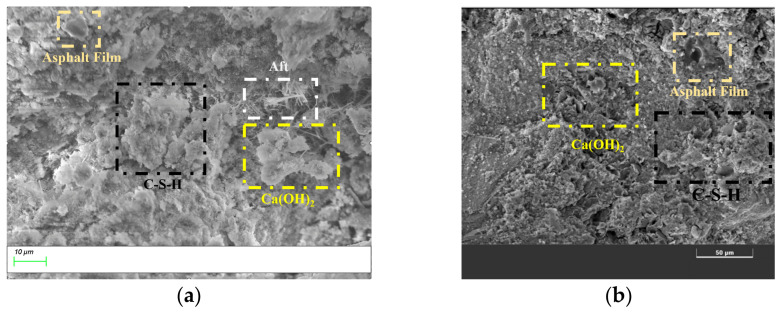
SEM scan results. (**a**) HMG-SFP. (**b**) SEM scan results of CMI-SFP.

Based on X-ray diffraction (XRD) phase analysis, scanning electron microscopy (SEM) was used to study the hydration products and gel structure of semi-flexible pavement mixtures prepared by different processes. Figure 13 shows the results of the microstructure of semi-flexible pavement mixtures.

As can be seen from Figure 13, a large number of hydration products are observed in the microstructure of SFP mixtures. SEM analysis showed that these products mainly include clustered calcium silicate hydrate (C-S-H), needle-like ettringite (AFt) crystals, and plate-shaped calcium hydroxide [Ca(OH)_2_]. These hydration products were intertwined with the asphalt film to form a three-dimensional network structure. The crystals, gels, and asphalt were closely combined, filling the internal voids of the mixture. The amount and morphology of hydration products varied with the preparation process, strongly influencing the microstructural characteristics of the material.

Under the CMI process, the hydration products were mainly composed of a large amount of flocculent and reticular C-S-H gels, which had good continuity in morphology and formed a dense encapsulation structure at the cement paste-aggregate interface. In contrast, in the HMG process, more acicular AFt crystals and sheet-like, loosely stacked Ca(OH)_2_ crystals were observed. In the CMI process, the encapsulation effect of emulsified asphalt on cement particles retarded the early hydration reaction to some extent and inhibited the rapid growth of AFt and the large-scale precipitation of Ca(OH)_2_, creating conditions for the secondary reaction of Ca(OH)_2_. This promoted its transformation into C-S-H gels and effectively filled the micropores on the aggregate surface and the interface transition zone. Compared to Ca(OH)_2_, the C-S-H gels exhibited better adhesion with the asphalt phase [30], which enhanced the adhesion strength between the binder and the aggregate and effectively prevented moisture ingress. Therefore, the CMI-SFP mixture showed better water stability at the macroscopic level, which was consistent with the aforementioned freeze–thaw splitting test results.

### 5.3. X-Ray Computed Tomography Testing and Analysis

**Figure 14:**
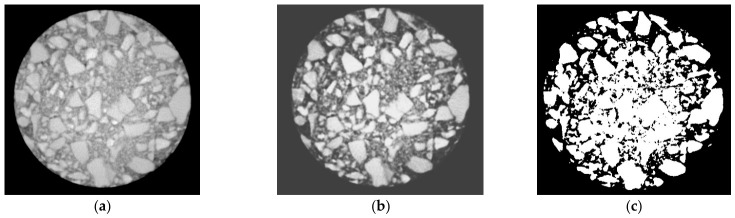
CT scanning cross-section image processing of SFPM (Sacle bar: 25 mm). (**a**) Grayscale image. (**b**) Image after equalization. (**c**) Multi-threshold segmentation.

**Figure 15:**
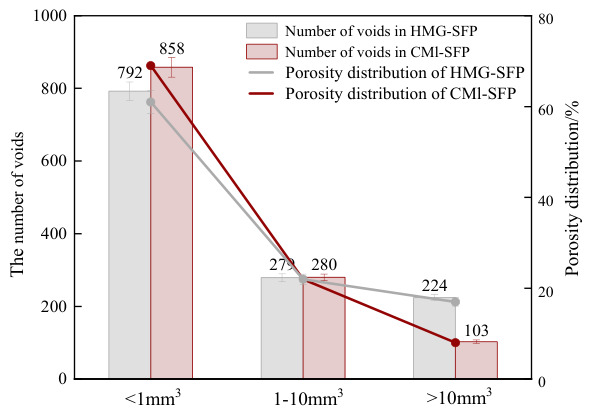
CT test results for SFP materials.

The CMI-SFP mixtures and HMG-SFP mixtures were prepared using standard Marshall specimens and rotary-compacted specimens with a 100 mm diameter in both cases. After curing for 7 days in a standard chamber, X-ray computed tomography (CT) was performed with layer-by-layer scanning. The CT images were reconstructed into three-dimensional models to analyze the vertical distribution of air void content and its variance within the pavement material. Pore identification and image processing were performed using MATLAB 2016a with the Image Processing Toolbox. Grayscale conversion, median filtering, and noise removal were applied to enhance image quality and pore recognition. The image processing procedure is shown in Figure 14. The grayscale histogram and the threshold value T, determined through iterative calculations, were used for mutual verification. Pore parameters were then extracted and statistically analyzed, and the pore rate distribution along the specimen height was finally obtained. Pore volumes were classified into three ranges: less than 1 mm^3^, 1–10 mm^3^, and greater than 10 mm^3^. Figure 15 shows the porosity distribution of voids.

Statistical analysis of their air-void content and variance along the height direction was then performed. Table 8 shows the summarized results.

Based on the CT scan results, it was observed that the overall void ratio of the CMI-SFP mixture was significantly lower than that of the HMG process, with a reduction of approximately 60%. This indicated that the process effectively improved the overall compactness of the material. Compared with the HMG process, the CMI process eliminated the traditional grouting step, avoiding the pores and local loose structures caused by insufficient grout penetration. This enabled the binder to be distributed more uniformly in the aggregate system, thereby forming a more continuous and stable spatial framework structure. In terms of void volume distribution, the proportion of micro voids with a volume less than 1 mm^3^ in the CMI mixture was approximately 8% lower than that of the HMG. Combined with the XRD analysis results, it was inferred that this process effectively filled the tiny pores in the material by promoting the formation and accumulation of C-S-H gel; in contrast, the proportion of large pores with a volume greater than 10 mm^3^ in the CMI-SFP mixture increased by approximately 8%, but these large pores were prone to stress concentration under low-temperature conditions, resulting in low-temperature cracking [31].

The statistical results of the surface void ratio along the height direction (Table 8) showed that the variance of the surface void ratio of the CMI-SFP mixture was approximately 90% lower than that of the HMG. This indicated that the integral preparation process significantly improved the uniformity of pore distribution along the height direction of the specimen. The smaller void ratio variance indicated a higher stability of the material’s internal structure, with a more uniform spatial distribution of asphalt, cement paste, and aggregates, thereby effectively reducing local vulnerable areas. Under repeated loading, this uniform and dense pore structure reduced [or could reduce] the initiation and expansion of micro-cracks, delayed the damage accumulation process, and thus improved the fatigue performance of the SFP mixture at the macroscopic level [32].

## 6. Conclusions

In response to the problems of uneven penetration and filling in the traditional HMG process, this paper proposes a CMI process. By systematically comparing the effects of different preparation processes and binder types on the performance and microstructure of the mixtures. Based on the above research, the following conclusions are drawn:

(1) Based on experimental research and performance evaluation, the influence of mixing sequence and compaction times on mixture design properties was studied, which led to the proposal of a cold-mixed integrated preparation process for semi-flexible pavement mixtures. This process eliminated the hot-mixing and grouting steps of traditional processes.

(2) SFP materials prepared with 70# base asphalt met specification requirements [20]. The addition of SBS-modified asphalt further improved their performance. Mixtures with SBS-modified emulsified asphalt performed best, showing approximately twice the improvement in high-temperature stability and a tenfold improvement in fatigue resistance compared to SBS-modified mixtures. SBS modification also enhanced low-temperature cracking resistance, but emulsification reduced it. The addition of SAE compensated for this drawback, increasing the low-temperature cracking resistance of semi-flexible pavement mixtures by about 7%.

(3) The CMI process enhanced the stiffness of the inorganic binder, which markedly reduced the variance of the void ratio of the specimens by approximately 90%. This effectively improved the uniformity of the material’s pore distribution, thereby significantly enhancing the high-temperature and fatigue life of the prepared material. Its dynamic stability was approximately 80% higher than that of the HMG and five times that of AC-13. The fatigue life exhibited an improvement of several orders of magnitude, with increases of approximately 200 times and 300 times compared to those of the HMG and AC-13.

(4) The CMI-SFP mixture exhibited superior water stability, S-SBS-CMI SFPM, with a residual strength improvement of approximately 7% compared to that of the S-SBS-HMG. One of the reasons for this was that the CMI process promoted the formation of C-S-H gel on the surface and within the interfacial transition zone (ITZ) of the aggregates. This effectively filled the micropores and enhanced the structural density, thereby inhibiting water infiltration and improving the material’s resistance to water damage.

(5) Future research should systematically clarify the coupled influence of emulsified asphalt dosage on the micro-macroscopic properties of the material. Specifically, it should investigate how different binder dosages regulate the evolution of the microstructure and interface characteristics, thereby establishing the intrinsic relationship between micro-properties and macroscopic mechanical behavior.

## Figures and Tables

**Table 1:** Matrix asphalt and SBS-modified asphalt technical specifications.

Test Materials	Penetration (100 g, 25 °C)	Softening Point (Ring-and-Ball)	Ductility 5 cm/min, 15 °C
Matrix asphalt	73	50	>150 (15 °C)
SBS-modified asphalt	46	69	>150 (15 °C)

**Table 2:** Modified emulsified asphalt technical specifications.

Technology Index	Test Values	Requirements
Demulsification rate	Slow	Rapid or medium cracking
storage stability/%	0.7	<1.0
Screen residue/%	0.02	<0.1
Residue content/%	60.5	≥55
Penetration/0.1 mm	70.2	45~150
Softening point/°C	46	≥42
Ductility	124	≥40

**Table 3:** SBS emulsified asphalt technical specifications.

Technology Index	Test Values	Requirements
Demulsification rate	Slow	Rapid or medium cracking
Viscosity/S	20	8–20
Screen residue/%	0.05	≤0.1
Evaporation residue solubility/%	50	≥50
Particle charge	No	Non-ionic (+)
Storage stability (5 d)	60	≥30

**Table 4:** Technical parameters of styrene–acrylic emulsion.

Test Content	Test Value
Appearance	Opalescent, blue-tinged liquid
pH value	7–8
Viscosity/Pa·s	500–2000
Solid content	45 ± 1
Film formation temperature/°C	25
Glass transition temperature/°C	35

**Table 5:** Cement mortar mix proportions and performance test results [19].

Water-to-Cement Ratio	Sand-to-Cement Ratio	Silica Fume-to-Cement Ratio	7 d		Flowability (s)	Compressive-to-Flexural Strength Ratio
			Compressive Strength (MPa)	Flexural Strength (MPa)		
0.6	0.1	0.15	24.5	6.9	11.9	1.64

**Table 6:** List of abbreviations for different semi-flexible pavement mixtures.

Abbreviation	Asphalt Type	Admixture	Preparation Processes
70-CMI material	70# emulsified asphalt	/	Cold-mixed integrated (CMI) process
SBS-CMI material	SBS-modified emulsified asphalt	/	CMI process
S-SBS-CMI material	SBS-modified emulsified asphalt	Styrene–acrylic emulsion (SAE)	CMI process
70-HMG material	70# base asphalt	/	Hot-mixed/grouted(HMG) process
SBS-HMG material	SBS-modified asphalt	/	HMG process
S-SBS-HMG material	SBS-modified asphalt	SAE	HMG process

**Table 7:** Marshall test results under different mixing methods.

Mixing Method	Compaction Times	Stability/KN	Flow Value/0.1 mm	Bulk Specific Gravity	Saturation/%
A	50	8.16	2.12	2.19	55.23
	75	8.79	2.10	2.16	57.80
B	50	8.19	2.23	2.17	55.40
	75	8.86	2.0	2.14	58.10

**Table 8:** Mean and variance of slice-wise air-void content from X-ray CT.

Preparation Process	Average Slice-Wise Air-Void Content	Slice-Wise Variance of Air-Void Content
CMI	0.03029	0.000011
HMG	0.03350	0.000125

## Data Availability

The original contributions presented in this study are included in the article. Further inquiries can be directed to the corresponding authors.
